# Data hygiene factors

**DOI:** 10.1016/j.patter.2021.100207

**Published:** 2021-02-12

**Authors:** Christopher Gutteridge

**Affiliations:** 1 University of Southampton, Southampton SO17 1BJ, UK

## Abstract

The goal of making your data available is that other people can reuse it. A number of factors can prevent anybody from ever exploiting your data. This article reviews some of these factors and suggests some low effort ways you can increase the chances of your data’s being used by others.

## Main text

Writing in the era of COVID-19, we’re all very aware of certain hygiene factors. Masks, distancing, handwashing. Hopefully we’re all aware that wearing 12 masks doesn’t make up for not washing our hands. In this article, I’m going to talk about ways to make your data more likely to be reused and the importance of doing everything to a minimum standard rather than overdoing some and neglecting others.

**Figure 1:**
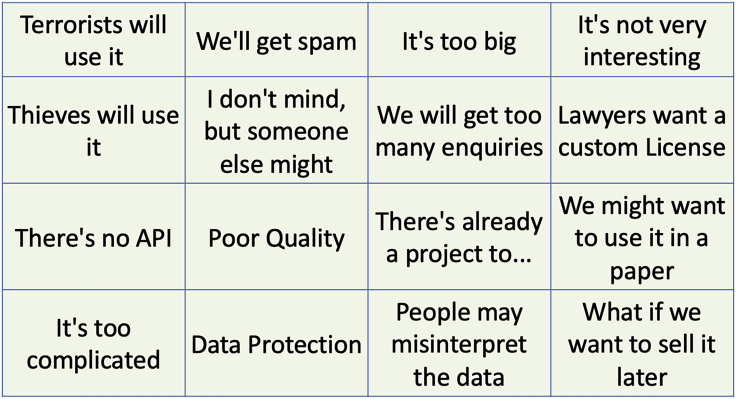
Bingo grid of reasons to not make data open

Where this started for me was a project to create open data about the University of Southampton.^1^ When we were publishing open data for the University of Southampton, we started noticing we were getting repeated reasons not to from different data owners. We turned this into a bingo grid to amuse future people following in our footsteps (Figure 1).

Where this became far more useful was at a talk I gave at the UK Open Data Institute (ODI) where I showed this slide and someone asked something such as “But what do you do when each of these happens? Surely you’ve solved some of these?” This inspired me and Alex Dutton (who was running the open data service at the University of Oxford) to create a document with these as headings and brainstorm what we’d learned. It turns out we knew lots of useful things we didn’t know we knew and the resulting document^2^ has been used and reused by people following in our footsteps.

A few years later, I worked with a student intern on a project to catalog entrances to University of Southampton buildings. I’d aimed to have a tickbox for “accessible.” This student was a wheelchair user himself and explained to me that there is no such thing as an “accessible” entrance. There are factors that make it inaccessible to some people. Very different factors make an entrance inaccessible to a wheelchair user, a blind person, and a person with epilepsy.

This applies to shared and open data too. There are no such thing as fully “open” datasets; there are factors that prevent their being reused. Such factors may be termed “hygiene factors.”

A hygiene factor is something that must never be low but that provides little extra benefit once it passes a threshold. Brushing your teeth once a week isn’t great but is still better than nothing. Brushing your teeth twice a day is good. Brushing your teeth every hour is far more work than twice a day for little extra benefit.

When we publish data with the hope it will be reused, we have a budget of effort we can realistically put in to increase the chances of its being reused. The problem is that, when things don’t work out, the temptation is to do even more of whatever we’re already doing rather than ignore all the things we’re not doing at all. This is equivalent to someone in the COVID-19 crisis who can’t be bothered to find out where to buy a facemask and so sanitizes their hands every 15 min. It’s wasted effort. The best results come from covering every factor enough, not from covering some a lot and others not at all.

FAIR^3^ (findability, accessibility, interoperability, and reusability) are the well-known factors and this ties in closely, but there are other factors to consider, including reputation and even rivalries and politics.

It is hard to know what factors we might have overlooked, so at Open Data Camp Belfast (2017), I ran an unconference session on the topic with about 40 people from the public sector who had experience publishing data that was reused (and not). Using what I had learned from making the open data excuses and solutions document, we brainstormed the reasons reuse fails to happen and what solutions the participants had found that actually worked. This was recorded in a “living” Google document^4^ that delegates worked on for some time after the event. Although our solutions were based on public sector open data, they translate well to research. Read the list and see whether there are any factors you’ve been ignoring or any that have not occurred to us.

Some of the factors that determine whether, and how much, your data is reused include value, audience, discovering, grasping, exploiting, perceived quality and reliability, and perceived neutrality.

Value and audience are more or less impossible to change unless you change your data itself. The other factors are things you have more control over, but valuable data with a large potential audience may still have a low uptake due to other factors’ being neglected.

### Value to audience

Almost any dataset has a potential value to some audiences but not all datasets are of equal value, of course. A 1924 Manchester tram timetable is less valuable than Blackbeard’s treasure map. You can relax about this factor as it’s intrinsic in the dataset you chose to make. (Please contact the author with your complaints listing scenarios where the tram timetable is more valuable at totl@soton.ac.uk.)

### Audience size

Closely related is how many people could benefit from this data if they were aware of it and could exploit it. There are not many ways to increase this either without changing the dataset. Making your dataset interoperable can increase the audience by making it part of something larger that’s useful to an audience when individual datasets are not.

### Ease of discovery

“If you build it, they will come” is a dirty lie. Making sure the data is discoverable with the correct search terms in the places your audience would be looking is a must. If it’s more unusual and people might not guess it exists, or there is no standard place to share it, then there is no shame in promoting the existence of your dataset to the potential audience. Get out there (or online during COVID-19) and build your network of peers. Another useful approach is to think of the search terms someone might use looking for something like this dataset and make sure all those terms are in your metadata.

### Ease of grasping the value

This is really important. If you fail to mention in the description the key features of value in your dataset, few will look at it even if it showed up in a set of results. A bit of empathy and marketing is needed. What is the audience for your dataset looking for in the summary?

Much like thinking about search terms, think about what your target audience is looking for and make sure you say those words. Writing articles about your dataset can help too. Peer reviewed papers are the gold standard, but a blog post about the dataset and why it’s interesting is a good idea. As ever, make sure you promote the post in appropriate ways such as personal social media. With skill, and some luck, people may share links on social media to peers who would be interested. Always make sure the metadata links to the blog post and the blog post links to the metadata.

### Ease of exploiting

Different people have different skills, which makes this a moving target, but you can help with documentation by using well-known standards from simple JSON/XML to domain specific ones, for example, code. One great suggestion from the community was to write a blog post about the dataset and link the dataset metadata to the blogpost and the blogpost to the dataset. However, the least effort to most gain suggestion was to include your electronic contact information—email or twitter handle. You can’t give detailed support to dozens of people, but the first person to try to reuse your data is special and you and they will both benefit from working together to get them started.

### Perceived quality and reliability

This factor is about trust and provenance. If people are just playing with ideas, they may not worry about this factor, but if they plan on using it for something more serious, then these matter. I’ve included the word “perceived” because it’s not just about the quality but also that people have faith in it. People don't know about your plans for an update schedule and your corrections policy unless you tell them*—*so clearly state these in the metadata and try to stick to them. For people making decisions based on your data, they want to be sure of where it came from and what assumptions and quality control were made in producing it. One thing we discovered in our open data project was that if a data source becomes temporarily unreliable, people stop using it for good. They move on and don’t come back once it’s fixed. Expectation management could help here; if there are downtimes or problems, make sure they are acknowledged and you have a way to communicate when they are resolved or else your data will be assumed to have “bitrot” (the degradation over time of an poorly maintained digital system).

### Perceived neutrality

Also known as “not invented here” syndrome. This is a difficult one and usually an issue when more senior people are in the mix. There is often a gentle steer away from using systems and data produced by organizations seen as rivals. This can sometimes be mitigated by reducing the organizational branding on the metadata of a dataset. It can also be mitigated by evangelizing your data to your peers at other organizations rather than their management.

### The unknown

There are almost certainly a few other factors we’ve failed to realize yet.

### Conclusion: quick wins

The overall message is that if you just produce a dataset and upload it to a repository, it might get reused, but there are lots of small chores you can do to significantly increase the chances of others’ getting some value from your work.

So, what is the quickest, cheapest way to remove barriers to people’s using your data? Based on the community experience, it is to identify one or more people who are the target audience and talk to them—ask them to be a critical friend. New eyes are a powerful tool, and chances are they will see at a glance things you didn’t think of, things that will be low effort to address.

Do the same for others, but if you do, don’t demand they do every agonizing detail of best practice; start with the things that would genuinely help you personally to find and use their dataset. That’s where their effort should be going.

A video discussing this topic is also available on YouTube at https://www.youtube.com/watch?v=3SvkOjEOCgc&feature=youtu.be&ab_channel=AI4ScientificDiscovery.

### Web resources

FI2NI: Giving your Open Data the best chance to realise its potential, https://www.youtube.com/watch?v=3SvkOjEOCgc&feature=youtu.be&ab_channel=AI4ScientificDiscovery

## References

[1] University of Southampton Open Data Service https://data.southampton.ac.uk/.

[2] Gutteridge C., Dutton A. Concerns about opening up data, and responses which have proved effective.. https://docs.google.com/document/d/1nDtHpnIDTY_G32EMJniXaOGBufjHCCk4VC9WGOf7jK4/edit.

[3] Wilkinson M.D., Dumontier M., Aalbersberg I.J., Appleton G., Axton M., Baak A., Blomberg N., Boiten J.W., da Silva Santos L.B., Bourne P.E. (2016). The FAIR Guiding Principles for scientific data management and stewardship. Sci. Data.

[4] Gutteridge C., Knight L., Crowther P., Braggins M., Thomas S., delegates at Open Data Camp (2017). Getting more value from Open Data publishing - Open Data Camp, Belfast 2017. https://docs.google.com/document/d/1vd8yOagTPPDcZsHpNMh-bIiKL3tjPDb4xrbXbP1hrdM/edit.

